# Footwear choices for painful feet – an observational study exploring footwear and foot problems in women

**DOI:** 10.1186/s13047-018-0265-2

**Published:** 2018-05-31

**Authors:** Moira McRitchie, Helen Branthwaite, Nachiappan Chockalingam

**Affiliations:** School of Life Sciences and Education, Staffordshire University, Leek Road, Stoke on Trent, ST4 2DF UK

**Keywords:** Footwear, Patient choices, Shoes, Painful feet, Footwear fit

## Abstract

**Background:**

A high percentage of the population report footwear related foot pain, yet there is limited research on the effect footwear has on the development of this pain. The aim of this study was to establish whether footwear purchased by patients have an association with foot pain and what choices determined a purchase decision.

**Methods:**

Shape and size measurements of the dominant foot and footwear (length and width) were taken from 67 female participants who routinely received podiatric treatment. Participants were also asked to complete a short questionnaire to rate the shoe characteristics, emotions whilst wearing and reasons for the purchase.

**Results:**

Results highlighted a high prevalence of structural foot pathology for those over 61 who preferred slip on shoes. This group also wore shoes that were significantly narrower than their feet with width difference correlating to the presence of Hallux Abductovarus (HAV). In addition, results indicate that individual footwear advice is more important than previously thought, as it is clear that choice of footwear worn to podiatry appointments are not always worn on a daily basis.

**Conclusions:**

This study emphasises that the width of the shoe is an important part of fit, highlighting the need for patient specific footwear assessment and education for behaviour changes.

## Background

Foot pathology and pain is reported in approximately 24–30% of the adult population with it being one of the top 20 reasons for seeing a doctor when over the age of 65 [1–4]. Foot pain has been associated with reduced mobility [5], decreased leg strength [6] and an increase in falls risk [7]. Ill-fitting footwear can increase foot pain, reduce stability inhibit relevant rehabilitation and increase hyperkeratotic lesions [8, 9]. Footwear characteristics such as heel height, toe box width, sole hardness and thickness have all been identified as elements that contribute to foot pain [10–12].

It is thought that habitual constriction caused by footwear causes osteological deterioration in feet over a long period of time [13], with unshod populations having a lower frequency of bony morphology [14]. However, in western populations there is a need to wear footwear to address environmental and functional requirements as well its role in identity [15], with young UK women purchasing on average 6 pairs of shoes a year [16].

The styling and fit of footwear worn can accelerate the chances of foot pain and the development of progressive foot deformity and pathology. Narrow toe boxes have been found to restrict the movement of the forefoot [17] resulting in a stiffer foot prone to increased stress from loading as well as significantly increasing dorsal and plantar forefoot pressures [18]. Fastening techniques used in shoe design have been shown to influence the normal width expansion of the shoes upper around the metatarsal heads, which if compressed increases internal stresses [19]. Similarly, the presence of a dorsal fastening on footwear improves ground clearance during gait and reduces the risk of falling [8]. Correct fitting of shoes also plays a role in pathology and pain with two thirds of feet measuring broader than the footwear chosen. This appears to be more prevalent amongst women who wear longer shoes than necessary to accommodate width or depth changes [10, 12, 20].

Footwear advice and prescription shoes, given as part of relevant rehabilitation related treatments, are often disregarded by individuals because of what the shoes look like [21]. The restricted choice of therapeutic and functional footwear has been linked to exclusion from activities, self-consciousness and vulnerability as well as lower self-esteem in individuals who are advised to utilise them as part of rehabilitation [22]. It has been proposed that women have an emotional relationship with their shoes [23] influenced initially by fashion and the need for personal identity before any considerations of pain and pathology. However, there are a number of other factors that affect purchase decisions and choices of footwear. Comfort and fit were the most important factors in the choice of shoes of an rheumatoid arthritis population [24] and footwear choices made by young women are often made related to the activity being undertaken [16].

Given that the number of shoe characteristics such as a narrow toe box [12] have previously been associated with pathology, it is essential to understand the reasoning behind the purchase decisions of a population with foot pain to then enable an effective rehabilitation intervention to be agreed. Therefore, the primary aim of this study was to establish which style of shoe were chosen by a female population who have independently sought podiatry treatment for foot pain, additionally identifying what factors influenced these footwear choices.

## Methods

Following Staffordshire University ethical approval, a sample was drawn from female patients who attend a private podiatric clinic in Cambridge UK, for routine podiatry care. Sixty-seven women were recruited during a 4-month summer time-period and informed consent was gained form all the participants. The inclusion of the women that were recruited were participants over 40 with a history of podiatric treatment for greater than 6 months. An observational study design was implemented to explore the choices made for footwear purchases as well as wearing habits. In addition to demographic and anthropometric data around foot pain and pathology.

### Data collection

A 4 point demographic questionnaire was used to gain foot shoe sizing measurements for each participant. This consisted of defining the shoe size of the dominant foot using a Brannock® (Liverpool, NY, USA) measuring device. Length was measured from the apex of the longest toe to heel and width was taken from the widest part of the forefoot at the circumference of the 1st and 5th metatarsal phalangeal joints. Measurements of the shoe worn to clinic were taken at the longest and widest part corresponding to the foot. Footwear was then categorised to styling and type [25]. Finally, a clinic assessment of the participant’s podiatric foot complaints related to ill-fitting footwear was made, defining joint deformity, hyperkeratoic skin lesions and participant’s presenting soft tissue pain.

A footwear choice questionnaire was then utilised to establish emotions about footwear worn, characteristics of the shoe and purchase influences when shopping for shoes. This multifaceted questionnaire has previously been validated for footwear choice [16] and follows 3 themes;

1. Footwear Fit – designed to collect data on shoe sizing, measurements, width fittings and advice taken on fit.
2. Footwear Purchases – Styling and type of shoe purchased including the number of shoes and reason for purchase.
3. Emotions associated with Footwear – Particularly how often each style of shoe is worn, comfort of shoe, negative and positive emotions as well as self-esteem

The questionnaire specifically explored information on shoe purchases over the previous 6 months. Consistency of the scales used for the questionnaire responses showed good internal reliability with Cronbach alpha coefficient reported for purchase influences of 0.854, characteristics of 0.927 and emotions of 0.719.

### Data analysis

Differences between each age group in foot length and width as well as shoe length and width were assessed with an independent-samples t-test.

Footwear categories were grouped into the following 6 styled types:

1. Slip-ons (mules, loafers and pumps),
2. Formal (court and dress shoes),
3. Open-toed (sandals and flip flops),
4. Boots,
5. Activity (trainers and walking shoes)
6. T-bars

Foot pathology was itemised as bone deformity (hallux adbucto valgus [HAV], hammer and claw digits), skin pathology (hyperkeratotic lesions, fissures and blisters) and soft tissue (musculoskeletal pathology, neuroma and plantar heel pain.) Differences for footwear worn to clinic and presenting symptoms were assessed with a Chi-square for independence.

Footwear choice data collected used a mix of continuous and categorical variables and was statistically analysed, using Cochrane’s Q test for footwear purchase choice and Kruskal-Wallis test for emotions. All data was analysed using the SPSS V24 (IBM) and set at 95%confidence (*p* > 0.05).

## Results

The recruited sample were split into two groups by age: 40-60 years old *Group A* and 61 plus years *Group B*. Demographic data for the two age Groups A and B is presented in Table 1. The older Group B were lighter and smaller in height than Group A and the foot was longer and narrower. Shoe sizing for older Group B was also significantly narrower than the younger Group A. There were no significant differences in footwear worn to clinic between the two age groups other than Group B were more likely to wear a slip-on shoe (Table 2).

**Table 1:** Group demographics for age (years), weight (Kg) height (cm) and foot size (cm). Differences between group A and B for foot size and shoe size are presented

		40-59 (*n* 32) Group A	61 + (*n* 35) Group B	*P* value A vs B
Age, y		52.8 +/− 5.9	74.2 +/− 8.5	–
Weight, kg		63.9 +/− 25.5	50.5 +/−32.1	–
Height, cm		152.9 +/− 43.3	121 +/− 72.4	–
Foot size, cm	Length	23.8 +/−3	24 +/−1	0.713
	Width	9.7 +/−2.9	9.5+/− 0.5	0.311
Shoe size, cm	Length	25.8 +/− 3.5	26.1 +/− 1	0.175
	Width	10.2 +/− 3.2	9.5 +/−0.5	0.04*

*p < 0.05 was considered statistically significant

**Table 2:** Frequency of participants footwear choices in each group A and B, for the Footwear category worn to clinic on the day of testing as well as the incidence of presenting symptoms

			40-59 (*n* 32) Group A	61 + (*n* 35) Group B	*P* value A vs B
Footwear Category	Slip - on		5	14^*^	0.047^*^
	Formal		3	1	0.261
	Open toed		9	9	0.824
	Boots		3	1	0.261
	Activity		10	7	0.29
	T-Bar		2	3	0.718
Presenting symptom	Bone deformity	HAV	12	23^*^	0.021^*^
		Hammer	13	9	0.194
		Claw	2	7	0.099
	Skin Pathology	Callus	29^*^	25	0.047^*^
		Corn	19	27^*^	0.013^*^
		Blister	1	0	0.292
	Soft tissue	Neuroma	2	1	0.502
		Heel pain	1	1	0.949

*p < 0.05 was considered statistically significant difference between group A and B

Between the two groups, the presenting symptoms associated with foot pain, highlighted significantly higher frequency of HAV and corns in Group B and the younger Group A were significantly more likely to present with a painful callus. The other presenting symptoms showed no significant difference between groups (Table 2). Further analysis of how fit transposed to incidence of painful pathology showed that 65% of presentation of HAVs were in shoes that were equal to or smaller in size than the corresponding foot.

**Fig. 1:**
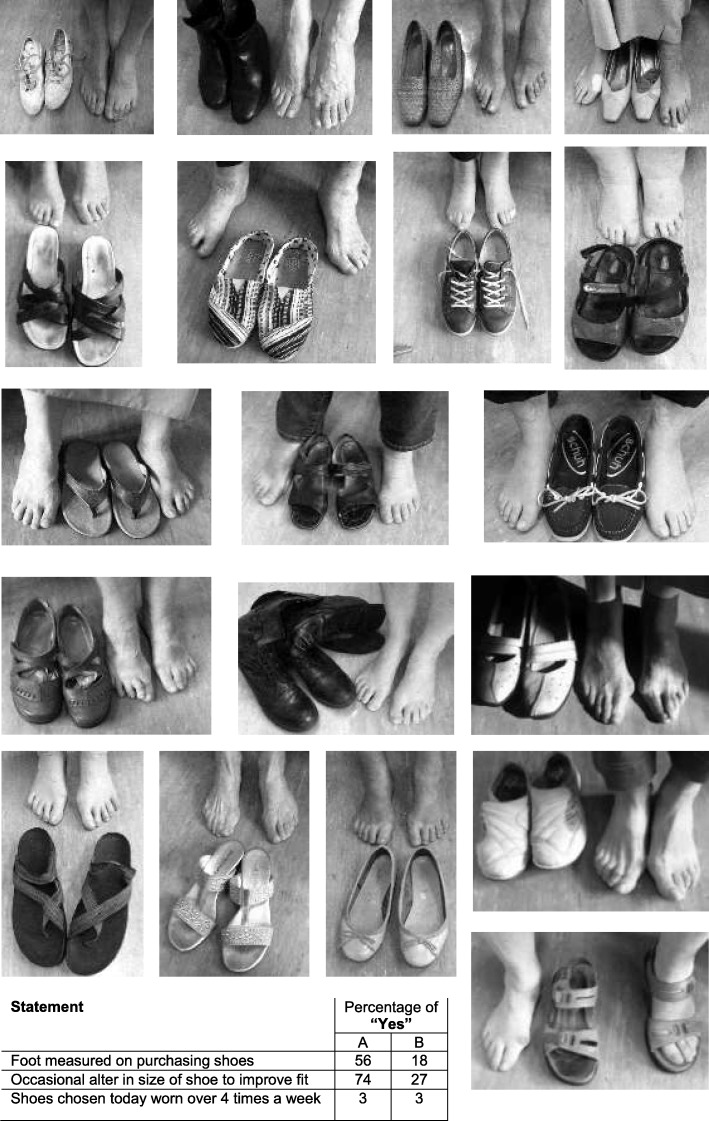
Types of footwear worn to clinic by a selection of participants with data on preferences of foot measurement, altering shoe size to improve fit and frequency of footwear worn

Participants were asked about their buying and wearing experiences of the shoes purchased in the previous 6-month period, the younger Group A were more likely to have feet measured prior to purchasing shoes and would alter the sizing of the shoe to fit compared to the older Group B who rarely had the foot measured and would not change the shoe size. Both groups wore the shoe chosen to attend clinic less than 4 times per week. Figure 1, illustrates the choice of footwear worn to the clinic from a randomly selected group of participants and clearly demonstrates the mismatch between the participants feet and their footwear and highlights the functional deficiencies of these footwear to support these participants.

**Fig. 2:**
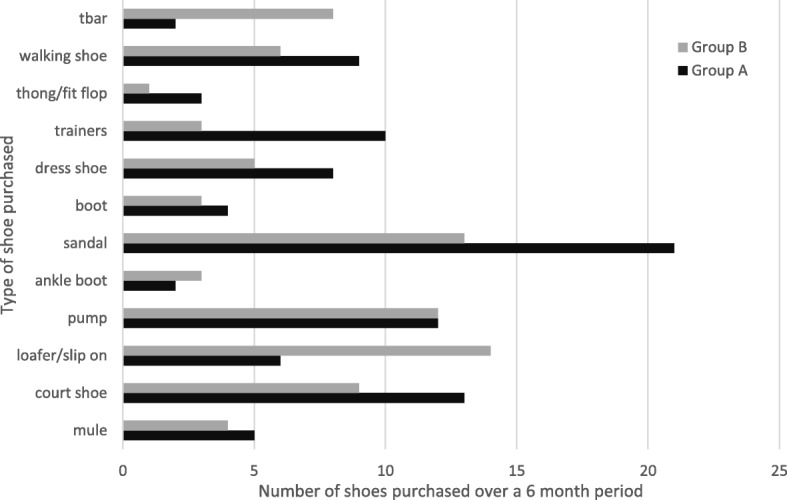
Number of shoes purchased by participants in Group A 40-60 years and Group B 61+ years, over a 6 month period categorised by footwear style

A grand total of 157 shoes were collectively bought over the 6-month period, Group A purchased 88 pairs, with sandals being chosen most frequently (*n* = 21) (Fig. 2). Purchasing factors that influenced choice of this type of shoe were comfort, summer holiday, and activity. Whilst for the older Group B bought fewer shoes (*n* = 69) were collectively purchased, with the slip-on shoe being most popular choice (*n* = 14) influenced by shape of heel, comfort, colour and fit (Cochrane Q, *p* = 0.000).

When exploring emotions related to purchase and wearing the selected shoes a statistically significant result was found using Kruskal Wallis test between how good patients felt about themselves and high levels of comfort (*p* = 0.031) and value of the shoe purchased (*p* = 0.009). Further Kruskal Wallis tests comparing these same qualities with width and length differences produced a statistically significant result (*p* = 0.030) between width difference and support only. When analysed by age group, no statistically significant results were found for the younger Group A (*p* = 0.126) but a statistically significant result was found for the Group B over 61 s (*p* = 0.049) with the median result indicating that there was a strong relationship between width difference (− 0.6 mm) and how comfortable the shoes were.

## Discussion

This study looked at the relationship between shoes purchased by a group of women receiving regular podiatry treatment and the presenting pathology, particularly whether shoes have an association with foot pathology and what choices determined the purchase decision. This group of participants, from a small locality of Cambridge UK, had independently sought podiatry treatment, as the presenting foot pain was perceived as requiring intervention.

The clinical presentations of foot pathology indicated a significantly higher incidence of HAV in the older group who were also more likely to have corns. It is known that morphological and physiological changes occur over time with bony and soft tissue changes occurring from natural ageing and prolonged mechanical strain [26]. The forefoot is reported to be wider with a greater depth in older adults and footwear that is not wide enough contributes to foot pathology [12]. However, in this study the older group had longer and narrower feet than the younger participants and did not consider having a foot measurement taken or change in footwear size when purchasing shoes. Therefore, any age-related changes that had occurred over time were not accounted for or considered by this group. This resulted in the shoe size worn to clinic being significantly narrower than the younger group with the shoe not fitting correctly to the measured size. There was no difference in width measurement from shoe or barefoot causing a tight fit of shoe. This could have led to an increase in incidence of ill-fitting footwear which is associated with the significantly higher incidence of corns formation. Further exploration of participants reasoning around these choices is warranted to investigate in greater detail the emotional behaviour associated with wearing footwear that doesn’t fit.

It is not uncommon for people to wear the wrong size of shoe; 60% of participants within both age groups from this study had a difference of more than 0.5 shoe size between right and left foot [27] with estimates of 86% wearing shoes that were narrower than their feet [20]. It is interesting to note that, in both groups, the shoes participants chose to wear to the clinic appointment were not worn for any more than 4 times a week and a variety of footwear styles were selected throughout the rest of the week. Branthwaite et al., (2012) indicated that the primary reason for footwear choice was the activity being undertaken and therefore the shoes, particularly the slip-on shoes, worn to clinic could have been selected for ease of removal in the clinic in preparation for the treatment. This is useful for clinicians completing a footwear assessment, as the results from this study suggest that footwear worn to clinic is often not the most used shoe and a thorough review of all shoes worn by an individual should be undertaken to give the most accurate and realistic advice about footwear choices. Exploring factors like footwear choice and other footwear styles worn will possibly help to reduce barriers between clinician and patient improving overall foot health and education [28].

Between the two age groups defined, there was no significant difference in the choice of footwear purchased over a 6-month period prior to data collection. However, slip on shoes were selected more frequently in the older age Group B than the younger Group A and sandals were a frequent choice for both groups but more so in the younger group. These results substantiate the findings of other research papers where the most common shoes worn by the elderly during the day were open toed shoes, slip on, sandals or slippers [24, 29]. These choices could be related to the climate and other environmental factors as the current study was conducted over the summer months and the previous referenced work was in the southern hemisphere, which may favour these types of footwear. Further work on the impact of seasonal footwear choice and foot pain will extend the understanding of the association between fit and styling of the shoes and presenting problems. This seasonal restriction needs to be considered when reviewing the results. However, slip on shoes could also be favoured by the older group over the younger participants as they are easier to put on and take off without the consideration for a fastening. Similarly, the locality of this single centre sample of participants could limit the generalisability of the observations made.

Although the heel height of a shoe is often suggested as a causative factor for HAV formation, with increases observed in forefoot plantar pressure and altered first ray function [12, 17, 30, 31], the results of the current study provide substantial evidence that age appears to be more important than previously thought in the formation of this joint deformity. However, it was not clear as to why participants with HAV chose to wear shoes that were smaller than the foot. There was a strong association between purchasing shoes and feel good factor, yet further exploration around the emotions around wearing smaller shoes was not investigated. Body image and a quest to hide deformity by choosing to wear normal fashion shoes could be responsible for this selection, as it is widely reported that orthopaedic shoes are deemed as ugly and often not worn [22].

A width difference of − 0.6 mm between the width of the foot and the shoe is significant to make a change in the comfort of the shoe. A comfortable good value shoe was considered important to make participants feel good and happy about themselves. Whilst it is commonly argued that a shoe is most comfortable when it mimics the shape of the wearers foot [32], the geometry of the forefoot which matches the shape of the toe box could be a critical factor in this opinion of comfort. However, often a shoe wide enough to fit the forefoot is not found in 66% of people [33] leading to the observed mismatch in footwear choice and foot dimension. This constraint and drawback with current footwear styling is stagnant and there is a clear need for improved understanding with possibilities for radical new last designs or innovative manufacturing of accommodative footwear uppers. With advances in technology relating to foot assessment and manufacturing techniques mass customisation of footwear is plausible. In addition, the development of 3D printing techniques makes it easy to provide patient specific footwear solution for effective clinical management. There were observed differences in the purchase decisions of these footwear between the two age groups. The younger group, when buying activity shoes, reported comfort, fit and support being the most important factors. This is similar to the previous work that suggested comfort and activity were the most significant factors that influence footwear purchases [16]. This was not observed in the older group who preferred a slip-on shoe with more fashionable factors of heel shape colour fit and comfort. This is suggestive that as women age, their body image is still of significant importance. This image is thought to play an important role in selection of fashion items regardless of age and disability [22].

To improve the level of compliance from a patient to clinical footwear advice a greater emphasis should be made on image and style of suggested footwear. Clinicians should be guided by patient’s choices and work to a realistic ideal to improve the success of footwear fit across all age groups. Individual discussions around patient choice and reasoning around footwear selection could improve understanding and influence behaviour of patients [28]. Individual education of the choices made and how that influences foot pain and pathology could improve the foot health of patients as well as influence fashion and image.

## Conclusion

Footwear assessment in clinical environments should consider the width of the shoe with greater scrutiny when explaining the relationship between ill-fitting footwear choice and foot pathology. In addition, the clinician should examine and review the range and variety of footwear worn by the individual patient. This enables the clinician to provide patient specific advice with appropriate consideration given to all types of footwear and activities of daily living rather than just looking at the shoes worn to clinic on the day of assessment which might not be the patient’s first choice.

## Abbreviation

HAV: Hallux abducto valgus
